# Corticocortical feedback increases the spatial extent of normalization

**DOI:** 10.3389/fnsys.2014.00105

**Published:** 2014-05-30

**Authors:** Jonathan J. Nassi, Camille Gómez-Laberge, Gabriel Kreiman, Richard T. Born

**Affiliations:** ^1^Department of Neurobiology, Harvard Medical SchoolBoston, MA, USA; ^2^Department of Ophthalmology, Boston Children's HospitalBoston, MA, USA; ^3^Swartz Center for Theoretical Neuroscience, Harvard UniversityCambridge, MA, USA

**Keywords:** visual cortex, corticocortical feedback, alert macaque, area summation, normalization

## Abstract

Normalization has been proposed as a canonical computation operating across different brain regions, sensory modalities, and species. It provides a good phenomenological description of non-linear response properties in primary visual cortex (V1), including the contrast response function and surround suppression. Despite its widespread application throughout the visual system, the underlying neural mechanisms remain largely unknown. We recently observed that corticocortical feedback contributes to surround suppression in V1, raising the possibility that feedback acts through normalization. To test this idea, we characterized area summation and contrast response properties in V1 with and without feedback from V2 and V3 in alert macaques and applied a standard normalization model to the data. Area summation properties were well explained by a form of divisive normalization, which computes the ratio between a neuron's driving input and the spatially integrated activity of a “normalization pool.” Feedback inactivation reduced surround suppression by shrinking the spatial extent of the normalization pool. This effect was independent of the gain modulation thought to mediate the influence of contrast on area summation, which remained intact during feedback inactivation. Contrast sensitivity within the receptive field center was also unaffected by feedback inactivation, providing further evidence that feedback participates in normalization independent of the circuit mechanisms involved in modulating contrast gain and saturation. These results suggest that corticocortical feedback contributes to surround suppression by increasing the visuotopic extent of normalization and, via this mechanism, feedback can play a critical role in contextual information processing.

## Introduction

Normalization has been advanced as a canonical computation in which a neuron's driving input is divided by the summed activity of a pool of neurons. This computational framework has been successfully applied throughout the visual system, across sensory modalities and in several different species (Carandini and Heeger, 2012). Response normalization has several attractive properties, particularly in sensory processing where it allows neurons with limited dynamic range to shift their response functions according to local statistics and reduces redundancy in neural population codes thus making them more efficient (Carandini et al., 1997).

Normalization has also been successful in explaining area summation properties in V1 (Cavanaugh et al., 2002), which are thought to contribute to a large spectrum of contextual effects in visual perception (Albright and Stoner, 2002). V1 neurons sum responses within the central region of their receptive field but display response suppression as stimuli invade the receptive field surround (Sceniak et al., 1999). Normalization can account for these non-linear responses, including the observation that reducing stimulus contrast increases the size of the summation field (Levitt and Lund, 1997). These effects of contrast on area summation properties are best captured by a model in which input drive and the normalization pool are stable in spatial extent but their relative gains depend on contrast (Cavanaugh et al., 2002).

Despite advancing our understanding of the phenomenological computations that subserve area summation, the actual neural mechanisms underlying normalization remain poorly understood. Circuits implementing normalization-like operations in V1 could be based on feed-forward signals combined with local non-linearities (Kayser et al., 2001), horizontal connections (Reynaud et al., 2012), or feedback signals from higher visual areas (Angelucci and Bressloff, 2006). While there is evidence that horizontal signals and, in particular, certain subtypes of interneurons are important in area summation properties (Adesnik et al., 2012), the extent to which feedback signals play a role is unclear. We recently showed that feedback from V2 and V3 contributes to surround suppression in V1 (Nassi et al., 2013): *inactivation* of feedback led to *increased* response magnitudes for large stimuli engaging the receptive field surround. The effect of feedback inactivation on surround modulation suggests that feedback may participate in normalization computations.

This study begins with the premise that area summation arises at least in part due to a divisive normalization mechanism and does not attempt to rule out alternative models. We sought to characterize how well a normalization model could account for feedback's influence on surround suppression and how different components of the normalization operation were affected by feedback and contrast. To do so, we applied a standard normalization framework to area summation data (reported by Nassi et al., 2013) and to new contrast response data obtained in V1 of the alert macaque with feedback from V2 and V3 intact or inactivated by cooling. We found that the observed decrease in surround suppression strength during feedback inactivation was best captured by a reduction in the spatial extent of a divisive normalization pool. On the other hand, the contrast sensitivity of area summation in the same neurons was better accounted for by changes in gain. We confirmed this parametric dissociation by showing empirically that (1) the summation field expansion caused by contrast reduction and (2) the contrast response function within the summation field both remain intact without V2/V3 feedback. These results support the idea that corticocortical feedback contributes to surround suppression by increasing the spatial extent of normalization independent of bottom-up drive and, thus, identify feedback as a key circuit element of a canonical computation operating throughout the brain.

## Materials and methods

All experiments were in accordance with the National Institutes of Health Guide for Care and Use of Laboratory Animals and were approved by the Harvard Medical Area Standing Committee on Animals.

### Experimental preparation and protocol

The experimental preparation and associated protocols have been described previously in Nassi et al. (2013). The details relevant to the current study are described here. Two male macaque monkeys (*Macaca mulatta*; M and R) were implanted with a head post and a scleral search coil for monitoring eye position. They were trained to fixate within a 1–1.5° window for 2–3 s for juice reward while seated in custom chairs (Crist Instrument). In each animal, three cryoloops were implanted in the lunate sulcus of the right hemisphere (Lomber et al., 1999) for reversible inactivation of V2 and V3 by cortical cooling, and a recording chamber (Crist Instrument) was implanted for electrophysiological access to V1 using tungsten microelectrodes (FHC). The extracellular recordings were acquired using a Cambridge Electronic Design 1401 system, and single- vs. multi-unit activity was determined using action potential waveform analysis software (Spike2).

Once a single-unit or multiunit cluster was isolated, we mapped the approximate borders of the receptive field [minimum response field (RF)], determined the optimal spatial and temporal frequencies, and preferred orientation/direction using a small grating. RFs were located at eccentricities between 2 and 6°, which were targeted so as to match the approximate retinotopy of regions in the lunate sulcus (V2/V3) in close proximity to the cryoloops. All subsequent tuning data were obtained using drifting sinusoidal gratings of mean luminance matching the surround (42 cd/m^2^) at the optimal direction and spatiotemporal frequencies, centered in the RF, with a 0.04° graded-contrast perimeter to reduce edge effects. Each stimulus presentation had a motion-onset delay of 250 ms (monkey M) or 300 ms (monkey R) after appearance and then moved for either 750 ms (monkey M) or 500 ms (monkey R). Tuning curves for the electrophysiology data were calculated from spike counts collected in the first 500 ms after motion onset. Between successive stimuli, we interposed 500 ms blanks at the mean luminance. Each stimulus condition was presented at least five times. The values of the parameter being varied in each experiment were always presented in block-wise random order.

Contrast response function data were obtained with sinusoidal gratings ranging in contrast from 0 to 99% in logarithmic steps. Area summation data were then obtained with sinusoidal gratings ranging in diameter from 0.125 to 8° (monkey M) or from 0.16 to 6.3° (monkey R) of visual angle in logarithmic steps. The full range of diameters was each presented at two different contrast levels, either in “block” design (monkey M) or randomly interleaved (monkey R). Differences in pre-stimulus firing rate (300–50 ms before stimulus onset) between contrast levels were not detected in either animal (Mann-Whitney *U*-test, each animal *p* > 0.1). The two contrast levels were chosen to span the linear portion of the contrast response profile (i.e., just above the spontaneous firing rate and just below 90% of the maximum firing rate).

This protocol was repeated while chilled methanol was pumped through the cryoloops, effectively ceasing all visually evoked activity in the visual field representation of V2 and V3 that corresponded to the V1 recording site. Pre-cooling (Control) and cooling (Feedback inactive) recording sessions ran for 40–60 min in total. Post-cooling area summation measurements were possible in 19 units, which confirmed the reversibility of reported effects. A series of control studies were performed in our previous study to rule out the possibility of direct cooling effects in V1 (Nassi et al., 2013). Critically, we observed no effect of cooling in the upper bank of the calcarine sulcus in V1, where neurons have receptive fields outside of the feedback “scotoma.” Furthermore, we could find no relationship between the magnitude of cooling effects and the proximity of recorded neurons to the V1/V2 border (details on experimental protocol and measurements of cortical temperature are found in Nassi et al., 2013).

### Area summation model

We analyzed the area summation data from each unit using a ratio of Gaussians model (ROG; Cavanaugh et al., 2002) whose response *R*_ROG_(*x*) is dependent on the stimulus diameter *x* ≥ 0. This model implements the canonical normalization computation described by Carandini and Heeger (2012), which can be written as:

(1) $$R_{\text{ROG}}(x)=R_{0}+\frac{D(x)}{\sigma +N(x)},$$

where *R*_0_ is the spontaneous rate, and σ > 0 is the constant background normalization pool activity. The model consists of two area summation components: *D*(*x*) ≥ 0 is the total excitatory drive, and *N*(*x*) ≥ 0 is the normalization pool input. Both *D* and *N* respond to a stimulus of diameter *x* ≥ 0 in proportion to its area under a Gaussian sensitivity function defined on the visual field. This form can be written in terms of the error function as

(2) $$\begin{array}{c} f(x)=\frac{1}{\sqrt{\pi }}\int_{-x/2}^{+x/2}e^{-{\left(\frac{y}{w}\right)}^{2}}dy\\ \;=w\cdot \text{erf}(x/2w), \end{array}$$

where the spatial extent for *D* and *N* are individually determined by the parameters *w*_*D*_ > 0 and *w*_*N*_ > 0, respectively. A stimulus of size *x* = *w* would lead to 52% of the area under this sensitivity function, making numerical estimates of *w*_*D*_ and *w*_*N*_ a practical benchmark of the drive and surround effective diameters. The underlying supposition is that summation relies heavily on the number of inputs from neurons with overlapping receptive fields: a large-*w* neuron receives more input than a small-*w* neuron and, thus, responds more vigorously over a larger visual field. Cavanaugh et al. (2002) had prescribed based on their data that both *D*(*x*) and *N*(*x*) be proportional to the square of the functions above, so that the model be explicitly written with subscripted gain parameters *k* and size parameters *w* as

(3) $$R_{\text{ROG}}(x)=R_{0}+\frac{k_{D}{\left[w_{D}\text{ erf}(x/2w_{D})\right]}^{2}}{\sigma +k_{N}{\left[w_{N}\text{ erf}(x/2w_{N})\right]}^{2}}$$

We set the constant σ = 1 and also found that it was necessary for stabilizing responses to small stimulus diameters (which make the normalization term in the denominator approach zero). To avoid local optima in equation (3), we fitted parameters in three nested stages. First, all parameters were constrained as 0 ≤ *k* < *K* and 0 < *w* < *W*, where the upper limits *K* and *W* were initially close to zero and progressively increased until the model fit mean-square-error (MSE) decreased to within 5% of the asymptotic MSE (obtained without upper limits). This favored solutions with smaller, interpretable, *k* and *w* values and avoided situations where parameters grew only to marginally improve MSE without any obvious changes in the fitted curve. Second, for each set of limits *K* and *W*, a grid of initial parameter values was used to identify the region where optimizations converge to the same solution. Third, we ensured that optimization steps were discretized according to the model's sensitivity to parameter changes, so that optimization steps in any parametric direction had equal effect on objective function output (the trust-region-reflective algorithm in Matlab's lsqcurvefit function can accomplish this by estimating the objective function's Jacobian matrix). Optimization was computationally demanding, so this routine was programmed for parallel processing on a high performance computer cluster.

### Area summation curve features

**Figure 1:**
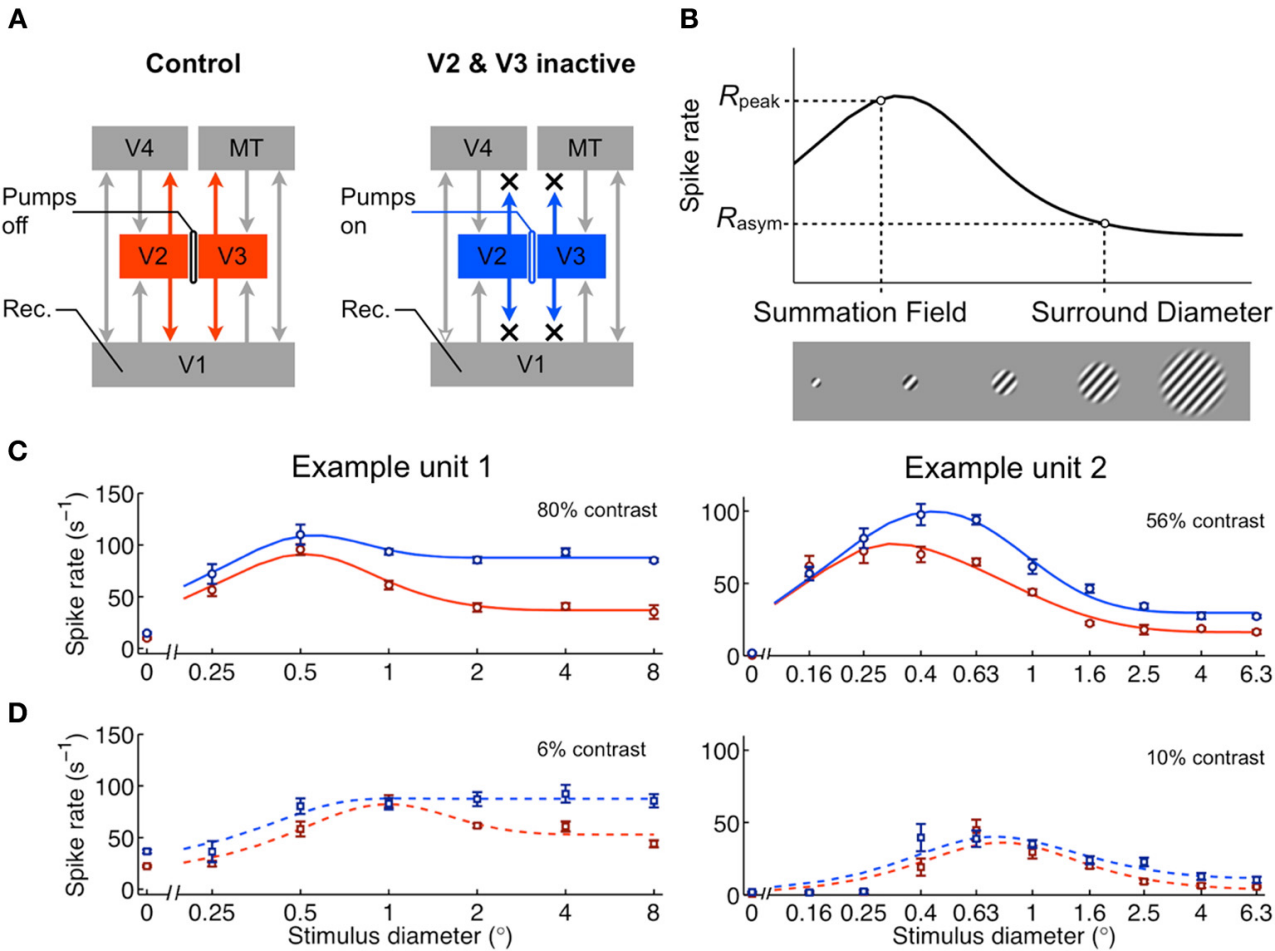
**Cortical feedback influences area summation in V1. (A)** Feedback to V1 was reversibly inactivated by cooling V2 and V3 using cryoloops (Materials and Methods). **(B)** Schematic of area summation curve measured using drifting gratings of various sizes, centered on the receptive field. The summation field, surround diameter, and their corresponding spike rates (*R*_peak_, *R*_asym_) are the main features characterizing the curve's shape. **(C)** Area summation curves from two example units during control (red) and feedback inactive (blue) conditions. Data are shown as mean ± s.e.m. (*n* = 5). The curves represent the fit from the model in Figure 2A. **(D)** Area summation curves measured from same units using low contrast gratings. The typical effect of feedback inactivation was an increase in response magnitude, particularly for high contrast gratings whose diameter extended beyond the summation field. The increased rate effect was present, yet diminished, during low contrast visual stimulation.

We defined the summation field size as the smallest diameter of the stimulus eliciting at least 95% of the peak response rate. Summation asymptote size was defined as the largest stimulus diameter that elicited a response 5% greater than the rate asymptote (when observable with largest diameters used). The strength of surround suppression was quantified with an index dependent on the response peak and asymptote spike rates: *SSI* = 1 − *R*_asym_/*R*_peak_, defined for units satisfying 0 < *R*_asym_ ≤ *R*_peak_. In the absence of surround suppression, *R*_asym_ = *R*_peak_ and so *SSI* = 0. As surround suppression becomes absolute (*R*_asym_ → 0; *R*_peak_ > 0), and so *SSI* → 1 (see Figure 1B).

### Contrast response model

Contrast sensitivity was quantified using the Naka-Rushton hyperbolic ratio function (Naka and Rushton, 1966) of the Michelson contrast 0 ≤ *c* ≤ 1 given by

(4) $$R(c)=R_{0}+R_{\max}\frac{c^{n}}{c^{n}+{\left(c_{50}\right)}^{n}},$$

where *R*_0_ and *R*_max_ are the measured spontaneous and maximum response rates, respectively, and *n* and *c*_50_ are the model parameters quantifying contrast sensitivity and contrast-at-half-response, respectively. This optimization problem was convex and was solved by least-squares minimization.

### Goodness-of-fit indices

These experiments required that the number of repeated trials for each data point be minimized (*n* = 5) in consideration of the large space of experimental conditions (i.e., grating size × contrast per baseline, cooling, and recovery block). Consequently, non-parametric methods like cross-validation were unattractive, since subsampled trials became too few to effectively average out across-trial fluctuations. We therefore opted to compare model configurations with equal number of parameters based on their percentage of variance explained. With an unequal number of parameters, we used a χ^2^_N_ statistic comparing model and measured rates, normalized by the optimization problem's degrees of freedom: *df* = *N*_stimuli_ − *N*_parameters_ + 1, where 1 is for the measured spontaneous rate (Hoel et al., 1971). Such parametric methods continue to be used in similar studies probing the effects of multiple experimental conditions on area summation (Carandini et al., 1997; Cavanaugh et al., 2002, 2012; Roberts et al., 2007; Olsen et al., 2010; Nienborg et al., 2013; Vaiceliunaite et al., 2013).

### Statistics

Values-in-text are reported as mean ± *SD* unless otherwise indicated. Goodness-of-fit comparisons and differential-responses to stimulus contrast, size, and feedback conditions were evaluated using ANOVA. Across-condition differences in summation features or spatial parameters (*w*) were evaluated with a two-tailed *t*-test. The gain parameters (*k*) were decidedly non-normal (spanning ~2 orders of magnitude) and, thus, were evaluated with the Mann-Whitney non-parametric *U*-test. The significance of Pearson correlation coefficient estimates *r* was determined following a *t* statistic conversion with H_0_: ρ = 0. Effects were considered significant for *p* < 0.05, and multiple simple-effect comparisons were accounted by Tukey's HSD test.

## Results

### Effects of feedback inactivation on area summation

We investigated how the area summation properties observed in V1 depend on corticocortical feedback from V2 and V3. To do so, we reversibly inactivated parts of V2 and V3 using “cryoloops” chronically implanted in the right lunate sulcus of two alert, fixating monkeys as reported in Nassi et al. (2013) (monkeys M and R; Figure 1A). Pumping chilled methanol through these loops cooled surrounding cortex down to temperatures below 20°C and allowed us to temporarily eliminate visually evoked activity across visuotopic regions of V2 and V3 overlapping and extending beyond the receptive field locations of recorded units in V1 (see Materials and Methods).

Area summation curves from 64 units (36 single units, 28 multi-units) in V1 were measured before and during inactivation of feedback by presenting drifting sinusoidal gratings centered within the receptive field and varying in size. The gratings were set to the preferred direction and spatiotemporal frequency for each unit. Due to the known effects of contrast on area summation (Sceniak et al., 1999), we tested effects of feedback inactivation at two contrast levels (“high” and “low;” see Materials and Methods). In this section, we analyze feedback inactivation effects at both contrast levels independently. In the subsequent section, we determine whether and how feedback inactivation interacts with the effects of contrast on area summation. All 64 units were sensitive to the size of the stimulus (Two-Way ANOVA, main effect stimulus diameter, each *p* < 0.05). In all cases the neural response initially increased as stimulus size increased from the smallest size tested (illustrated schematically in Figure 1B), peaked for stimuli of intermediate size and were almost always reduced for larger sizes. The observed response profile—characterized by initial summation followed by surround suppression—is typical for neurons in V1 (Sceniak et al., 1999). Feedback inactivation altered the overall response magnitude in 40 of 64 units (main effect feedback, each *p* < 0.05), increasing responses on average, and interacted with stimulus diameter in 29 of 64 units (diameter × feedback interaction, each *p* < 0.05). As reported previously, the most common effect of inactivation was an increase in response magnitude, particularly for grating diameters that extended beyond the borders of the receptive field center (Figure 1C) (Nassi et al., 2013).

**Figure 2:**
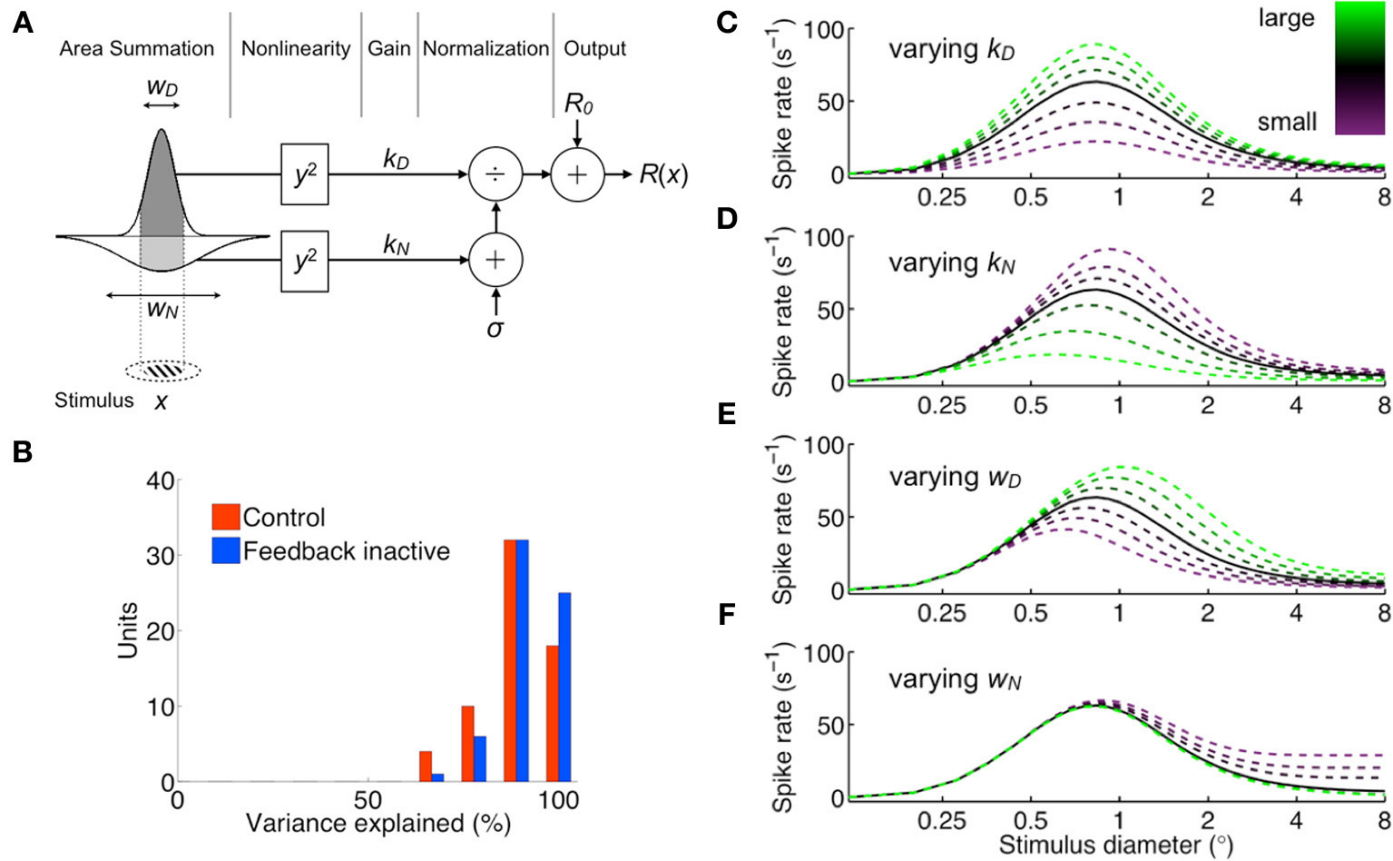
**Normalization area summation model (ratio of Gaussians). (A)** Schematic representation of the model. The receptive-field-centered grating stimulus of diameter *x* determines the integration bounds of the excitatory drive (dark shading) and normalization pool (light shading) area summation components, characterized by spatial parameters *w*_*D*_ and *w*_*N*_, respectively. Their outputs are squared and amplified by gains *k*_*D*_ and *k*_*N*_ before normalization. The constant σ represents baseline normalization pool activity and is set to unity without loss of generality. *R*_0_ is the measured spontaneous rate, and *R*(*x*) is the response spike rate. **(B)** Summary of model goodness-of-fit per unit for both control (red) and feedback inactive (blue) conditions (median variance explained: 90% control, 93% feedback inactive). **(C–F)**, Illustration of area summation curve effects due to individual variation of each model parameter. Population-averaged parameters produce solid black curve, and the effects of increasing/decreasing one parameter are plotted as green/purple broken curves.

In order to investigate the underlying mechanisms that give rise to the observed effects of feedback inactivation, we fit the area summation data with the “ratio of Gaussians” (ROG) model (Cavanaugh et al., 2002), a form of divisive normalization that implements an excitatory drive component *D*(*x*) ≥ 0, dependent on the stimulus diameter *x*, whose output is divided by the diameter-dependent activity *N*(*x*) ≥ 0 and background activity σ > 0 of the unit's normalization pool (Figure 2A). Under the assumptions of this model, it is possible to infer the response gain and visual field extent of the excitatory drive {*k*_*D*_, *w*_*D*_} and normalization pool {*k*_*N*_, *w*_*N*_} components accounting for receptive field center-surround interactions. We fit the model to the responses of each unit independently by allowing all parameters to vary for both feedback conditions. This unbiased approach is best when probing for a link between an experimental effect and a parameter, provided that measures are taken to avoid redundancy/trade-offs between parameters as well as to counter-balance potential differences in parameter sensitivity (see Materials and Methods). Potential links between experimental conditions and parameters were further tested by comparing constrained models. Although the number of parameters was large relative to the data points (8 parameters for 12–18 data points), each parameter's influence on the area summation curves was well constrained—only *k*_*N*_ and *w*^−1^_*D*_ had qualitatively similar influences, yet even in this case *k*_*N*_ primarily affects peak rate and *w*_*D*_ primarily summation field size (Figures 2D,E). This version of the normalization model was shown to be optimal in accounting for similar data in anesthetized macaques (Cavanaugh et al., 2002). The ROG model accounted well for the area summation data with and without feedback, explaining 90% of the variance overall across the population both in the feedback and control conditions (Figure 2B, see example fits in Figures 1C,D).

**Figure 3:**
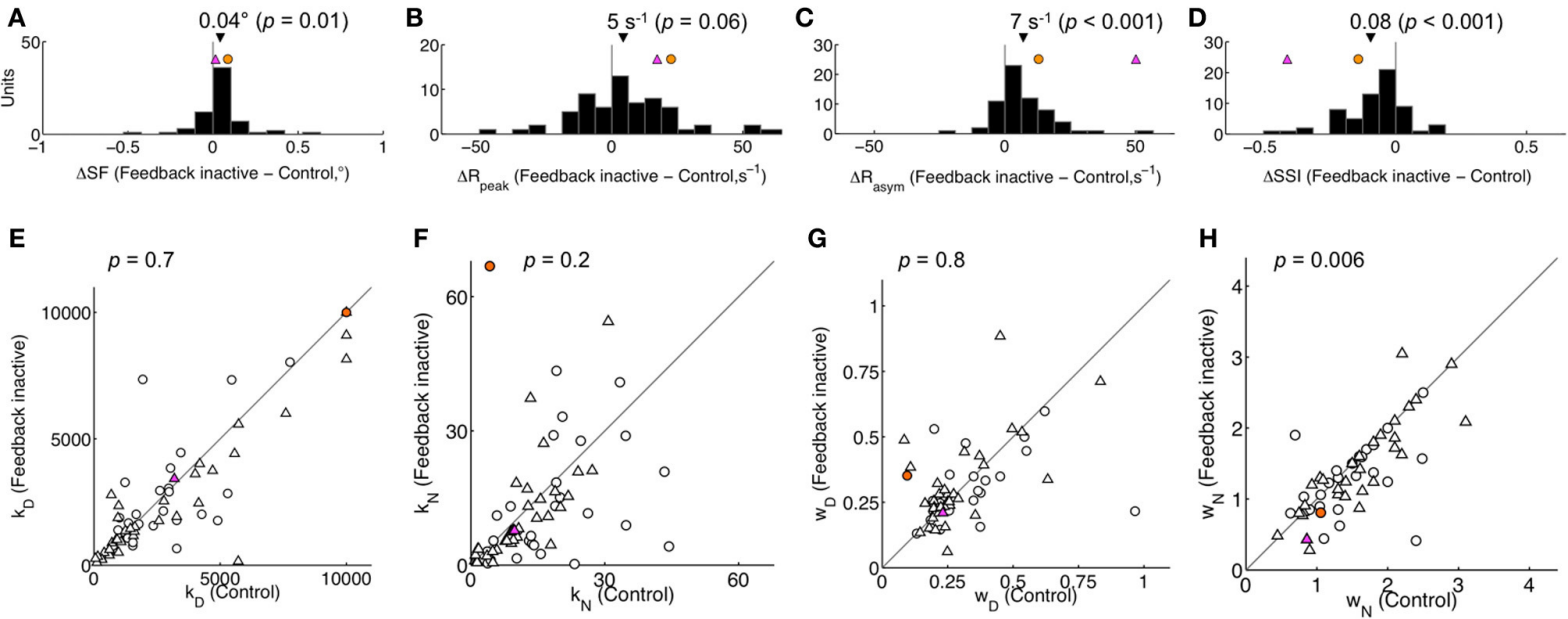
**Population effect of feedback inactivation on high contrast area summation. (A–D)** Summation curve features: difference in summation field (SF), peak response rate (*R*_peak_), asymptote response rate (*R*_asym_), and surround suppression index (SSI) between feedback inactive and control conditions. Black triangle indicates population mean (with two-tailed *t*-test *p*-value). **(E–H)** Parameter estimates (*k*_*D*_, *k*_*N*_, *w*_*D*_, *w*_*N*_) for all units plotted for Feedback inactive vs. Control conditions. *p*-values correspond to a two-tailed Mann-Whitney *U*-test for *k*_*D*_ and *k*_*N*_, and a *t*-test for *w*_*D*_ and *w*_*N*_. Triangles and circles indicate single- and multi-units, respectively. Differences were not detected in any feature **(A–D)** or parameter **(E–H)** when comparing single- and multi-unit subpopulations (each *p* > 0.05). In all panels, the magenta and orange symbols indicate example units 1 and 2 in Figure 1. Feedback inactivation reduced SSI by increasing *R*_asym_. The model accounted for this effect by systematically reducing *w*_*N*_ across the population.

From the fitted area summation curves, we measured several features (illustrated in Figure 1B): the summation field diameter (SF), the surround diameter and the respective peak and asymptotic response rates (*R*_peak_, *R*_asym_). Each of these features was compared across the population for control and feedback-inactivated conditions (Table 1). For high contrast stimuli (Figures 3A–D), the most prominent effect of feedback inactivation was an increase in *R*_asym_ of 7 spikes/s on average (67% increase from control; *p* < 0.001; Figure 3C). This caused the surround suppression index (SSI) to fall on average by 8% (*p* < 0.001; Figure 3D). For low contrast stimuli, asymptote rates increased as well; however, a concomitant increase in peak response rates resulted in only a 3% reduction in SSI (*p* = 0.10; Table 1). We also observed additional effects during high contrast stimulation: an increase in SF of 0.04° on average (*p* = 0.01; Figure 3A) and an average decrease in surround diameter of 0.3° (*p* = 0.009; Table 1). These latter changes, however, were small in comparison to the reduction in surround suppression observed for high contrast stimuli. Together, these results indicate that the main effect of feedback inactivation on area summation in V1 is to increase responses to large stimuli and therefore reduce the overall strength of surround suppression (Nassi et al., 2013).

**Table 1:** **Feedback's effect on area summation curve features and model parameters**.

**High contrast**	**Control**	**Feedback inactive**	***p*-value**
Summation field diameter (°)	0.46 ± 0.20	0.51 ± 0.22	0.01
Peak response rate (s^−1^)	54 ± 32	59 ± 35	0.06
Surround diameter (°)	4.4 ± 1.5	4.1 ± 1.6	0.009
Asymptote rate (s^−1^)	12 ± 15	19 ± 20	<0.001
SSI (a.u.)	0.79 ± 0.17	0.71 ± 0.22	<0.001
*k*_*D*_ (a.u.)	1730 [3110]	1770 [2440]	0.7
*k*_*N*_ (a.u.)	12.9 [13.8]	7.99 [14.5]	0.2
*w*_*D*_ (°)	0.32 ± 0.25	0.31 ± 0.18	0.8
*w*_*N*_ (°)	1.96 ± 1.29	1.72 ± 1.22	0.006
**Low contrast**	**Control**	**Feedback inactive**	***p*-value**
Summation field diameter (°)	0.71 ± 0.65	0.72 ± 0.38	0.9
Peak response rate (s^−1^)	32 ± 22	37 ± 27	0.01
Surround diameter (°)	4.6 ± 1.6	4.7 ± 1.5	0.6
Asymptote rate (s^−1^)	9.6 ± 12	12 ± 14	0.01
SSI (a.u.)	0.71 ± 0.26	0.68 ± 0.25	0.10
*k*_*D*_ (a.u.)	532 [613]	450 [817]	1
*k*_*N*_ (a.u.)	5.02 [5.89]	3.79 [5.82]	0.1
*w*_*D*_ (°)	0.39 ± 0.29	0.40 ± 0.20	0.8
*w*_*N*_ (°)	2.14 ± 1.31	2.11 ± 1.33	0.6

Mean ± SD and t-test used everywhere except for k_D_ and k_N_ reported as median [IQR] and Mann-Whitney U-test.

To gain insight into the mechanisms underlying the effects of feedback inactivation, we examined how the variation of each model parameter influences the area summation curve (Figures 2C–F). These figures show that the normalization pool's spatial extent *w*_*N*_ alone could account for the effect of feedback inactivation, since reducing *w*_*N*_ leads to an increased response for large stimuli extending into the receptive field surround (i.e., increased *R*_asym_), while leaving the responses to small stimuli confined to the center unchanged (Figure 2F). By contrast, changes in the gain parameters (*k*_*D*_, *k*_*N*_) and the excitatory drive's spatial extent (*w*_*D*_) are unable to affect responses to large stimuli without also affecting responses to smaller stimuli (Figures 2C–E). Not only do the other parameters primarily affect *R*_peak_, but *k*_*N*_ and *w*_*D*_ also tend to produce sizeable shifts in SF, both of which were not typically observed in our data. When we compared the parameter estimates from the model across feedback conditions at high contrast, *w*_*N*_ was significantly reduced during feedback inactivation from 1.96 ± 1.29° to 1.72 ± 1.22° (mean ± *SD, p* = 0.006; Figure 3H). The reduction in *w*_*N*_ was not observed for low contrast stimulation (2.14 ± 1.31° vs. 2.11 ± 1.33°, *p* = 0.6; Table 1). This is not surprising given the much weaker effects of feedback inactivation at low contrast. Nevertheless, we did detect a population-wide relationship between feedback-induced changes in *w*_*N*_ and SSI for both high and low contrast conditions (*r*_high_ = 0.30, *r*_low_ = 0.29, each *p* < 0.05). In agreement with Figures 2C–F, the remaining parameters *k*_*D*_, *k*_*N*_, and *w*_*D*_ showed no trend of systematic change during feedback inactivation at either of the two contrast levels tested (Mann-Whitney *U*-test for *k*, and *t*-test for *w* parameters, each *p* > 0.1; Figures 3E–G).

According to the area summation model results (Figure 3), the effects of feedback inactivation on area summation were best captured by changes in the normalization pool's spatial extent (*w*_*N*_). In order to further determine the individual contributions of each parameter, we compared versions of the model in which only one of the four parameters was fixed across feedback conditions and the other three parameters could freely vary. Only small reductions in percentage of variance explained were observed when fixing any single parameter, primarily because all models captured the main curve features well. Nevertheless, we detected significant differences among these four fixed-parameter models (One-Way ANOVA, main effect of model, *p* = 0.001; Table 2). Fixing *w*_*N*_ resulted in the worst fits to the data (Tukey's test on fixed-*w*_*N*_ simple effects, all *p* < 0.05), while differences in fit quality were not detected for the three remaining fixed-parameter models (main effect excluding fixed-*w*_*N*_ model, *p* = 0.8). The differences among the fixed-parameter models underscore that changes in *w*_*N*_ were important to capture the effects of feedback inactivation. In the context of the normalization model, our results indicate that the effects of feedback inactivation on area summation are best explained by a reduction in the spatial extent of the normalization pool.

**Table 2:** **Model comparisons across feedback conditions**.

	**Area summation**	***k_D_* fixed**	***k_N_* fixed**	***w_D_* fixed**	***w_N_* fixed**
Variance explained (mean ± *SD*)	90 ± 8%	87 ± 7%	86 ± 9%	87 ± 9%	82 ± 11%
One-Way ANOVA (main effect):					*p* = 0.001
Tukey's (simple effects paired with *w*_*N*_ fixed):					*p* < 0.05
One-Way ANOVA (main effect without *w*_*N*_ fixed):					*p* = 0.8

Variance explained by the area summation model and the four versions that fix one parameter across feedback conditions.

### Contrast gain mechanism unaltered during feedback inactivation

**Figure 4:**
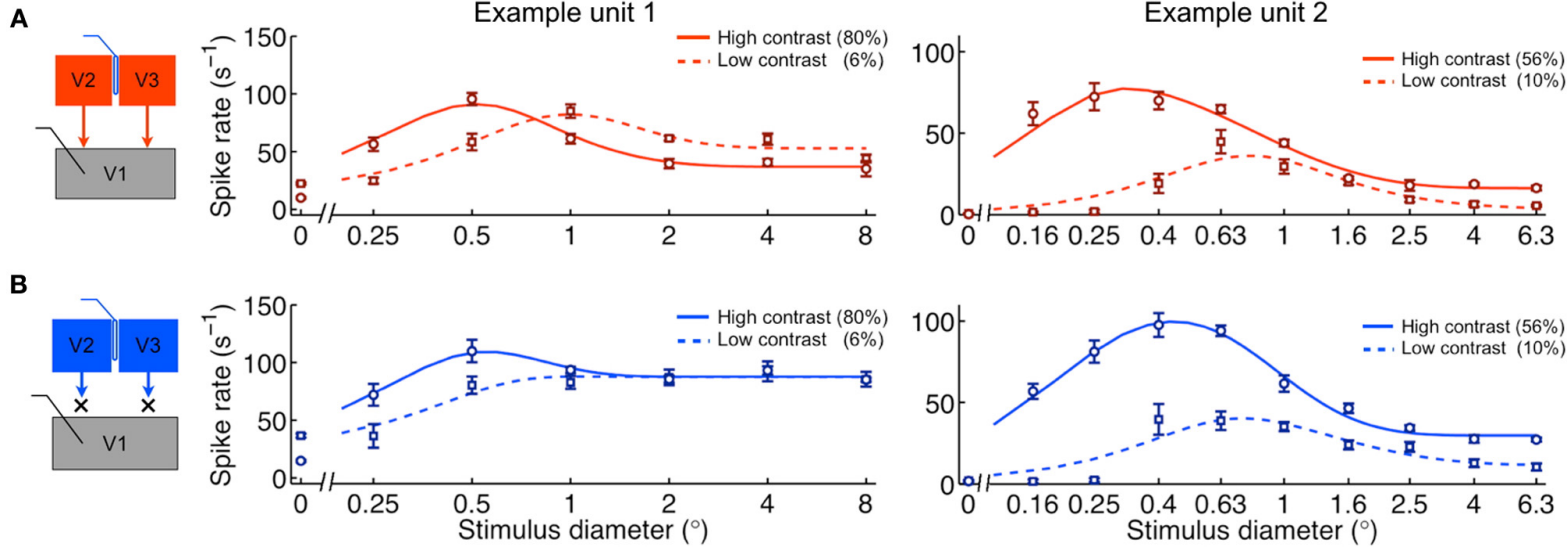
**Contrast effects on V1 area summation persist during cortical feedback inactivation. (A)** Area summation curves during control condition for same example units in Figure 1. **(B)** Area summation during feedback inactivation. The typical effect of lowering contrast is a reduction in peak response rate and an increase in summation field. This effect persists without feedback from V2 and V3.

**Figure 5:**
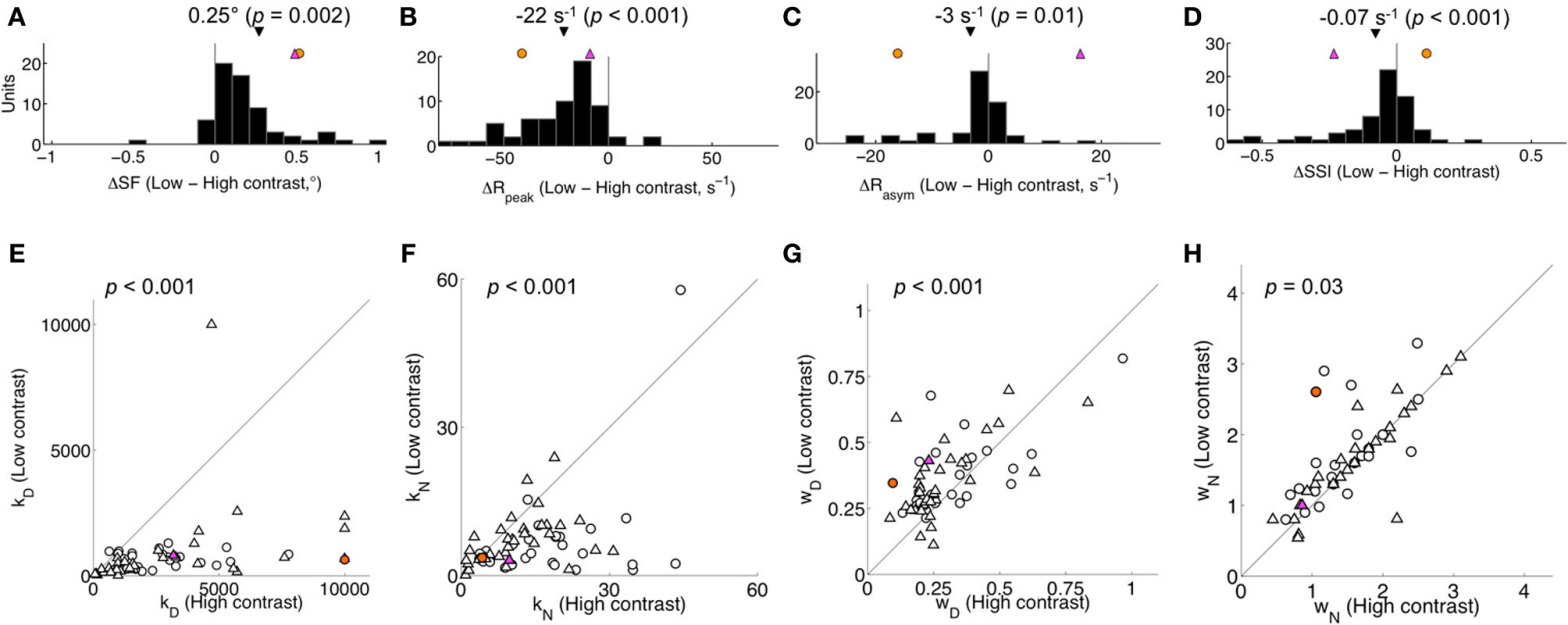
**Population effect of stimulus contrast on area summation during the control condition. (A–D)** Summation curve features: difference in summation field (SF), peak response rate (*R*_peak_), asymptote response rate (*R*_asym_), and surround suppression index (SSI) between high and low contrast stimuli. Black triangle indicates population mean (with two-tailed *t*-test *p*-value). **(E–H)** Parameter estimates (*k*_*D*_, *k*_*N*_, *w*_*D*_, *w*_*N*_) for all units plotted for high contrast vs. low contrast conditions. *p*-values correspond to a two-tailed Mann-Whitney *U*-test for *k*_*D*_ and *k*_*N*_, and a *t*-test for *w*_*D*_ and *w*_*N*_. Triangles and circles indicate single- and multi-units, respectively. Differences were not detected in any feature **(A–D)** or parameter **(E–H)** when comparing across single- and multi-unit subpopulations (each *p* > 0.05). In all panels, the magenta and orange symbols indicate features for example units in Figure 1. Reducing contrast increased the summation field, decreased the peak response rate, and yielded a small decrement in the asymptotic rate and suppression index. Both gain parameters increased and *w*_*D*_ decreased with high contrast.

**Figure 6:**
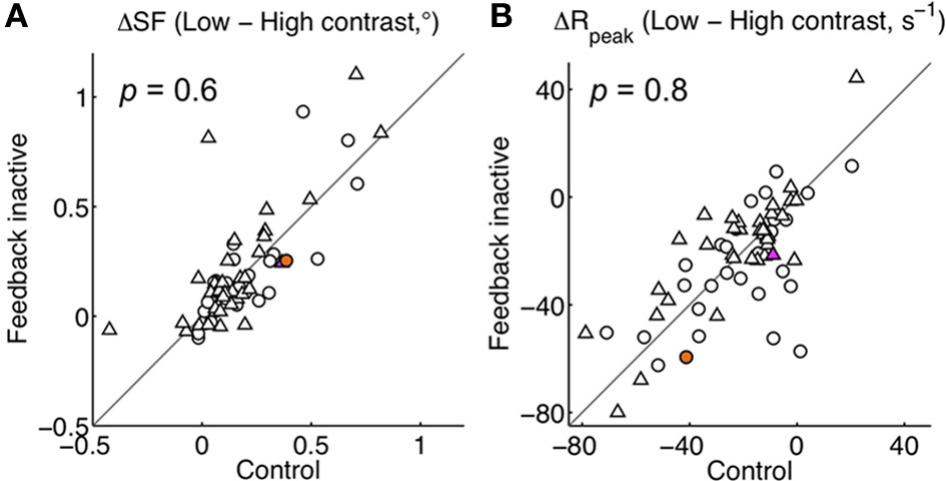
**Contrast-induced effects remain largely intact during feedback inactivation. (A)** Shift in summation field from high to low contrast (ΔSF) plotted per unit for Feedback inactive vs. Control conditions. **(B)** Corresponding shift in the peak response rate (Δ*R*_peak_). For both panels *p*-values correspond to a two-tailed *t*-test between feedback conditions. The magenta and orange symbols indicate shifts for the example units in Figure 1. No systematic change in contrast-induced effects were detected when feedback was inactivated.

The above analysis suggests that feedback inactivation had little or no effect on the gains of the underlying excitatory drive and normalization pool components. In order to test this possibility further, we analyzed the effects of stimulus contrast on area summation, both with and without V2/V3 feedback, which have previously been shown to be mediated by changes in gain (Cavanaugh et al., 2002). If feedback effects are indeed independent of these gain mechanisms, we would expect the effects of stimulus contrast to be unaltered during feedback inactivation. We found that contrast had a significant effect on the area summation data for the majority of the population both with feedback intact (55 of 64 units) and inactivated (47 of 64 units) (Two-Way ANOVA, diameter × contrast interaction, each *p* < 0.05). Consistent with previous reports, the typical effect observed when lowering contrast was an expansion of the summation field and a reduction in peak response rate (Figure 4) (Sceniak et al., 1999). These effects were observed independently of feedback state. We quantified these effects at the population level using the area summation curve features obtained from the area summation model (Table 3). During the control condition, we confirmed that reducing stimulus contrast significantly increased SF from 0.46 ± 0.20° to 0.71 ± 0.65° (*p* = 0.002; Figure 5A) and significantly decreased *R*_peak_ from 54 ± 32 s^−1^ to 32 ± 22 s^−1^ (*p* < 0.001; Figure 5B). The magnitude of these contrast-induced shifts in summation field diameter and peak response rate did not differ between feedback conditions (ΔSF: −0.25 ± 0.60° vs. −0.21 ± 0.28°, Δ*R*_peak_: 22 ± 20 s^−1^ vs. 22 ± 21 s^−1^, feedback on vs. off respectively, each *p* > 0.1; Figures 6A,B), suggesting that the effect of contrast on area summation remained largely intact in the absence of feedback. In addition, there was a smaller contrast-induced effect on *R*_asym_ (Figure 5C). The contrast-induced decrease in *R*_asym_ was larger during feedback inactivation than during the control condition. This asymmetry explains why reducing stimulus contrast significantly decreased SSI during the control condition (average 8% decrease, *p* < 0.001; Figure 5D) but not during feedback inactivation (average 3% decrease, *p* = 0.14; Table 3).

**Table 3:** **Contrast's effect on area summation curve features and model parameters**.

**Control condition**	**High contrast**	**Low contrast**	***p*-value**
Peak size (°)	0.46 ± 0.20	0.71 ± 0.65	0.002
Peak rate (s^−1^)	54 ± 32	32 ± 22	<0.001
Asymptote size (°)	4.4 ± 1.5	4.6 ± 1.6	0.09
Asymptote rate (s^−1^)	12 ± 15	9.6 ± 12	0.006
SSI (a.u.)	0.79 ± 0.17	0.71 ± 0.26	<0.001
*k*_*D*_ (a.u.)	1730 [3110]	532 [613]	<10^−10^
*k*_*N*_ (a.u.)	12.9 [13.8]	5.02 [5.89]	<10^−5^
*w*_*D*_ (°)	0.32 ± 0.25	0.39 ± 0.29	<0.001
*w*_*N*_ (°)	1.96 ± 1.29	2.14 ± 1.31	0.03
**Feedback inactive**	**High contrast**	**Low contrast**	***p*-value**
Peak size (°)	0.51 ± 0.22	0.72 ± 0.38	<0.001
Peak rate (s^−1^)	59 ± 35	37 ± 27	<0.001
Asymptote size (°)	4.1 ± 1.6	4.7 ± 1.5	0.001
Asymptote rate (s^−1^)	19 ± 20	12 ± 14	<0.001
SSI (a.u.)	0.71 ± 0.22	0.68 ± 0.25	0.14
*k*_*D*_ (a.u.)	1770 [2440]	450 [817]	<10^−8^
*k*_*N*_ (a.u.)	7.99 [14.5]	3.79 [5.82]	<0.001
*w*_*D*_ (°)	0.31 ± 0.18	0.40 ± 0.20	<0.001
*w*_*N*_ (°)	1.72 ± 1.22	2.11 ± 1.33	0.003

Mean ± SD and t-test used everywhere except for k_D_ and k_N_ reported as median [IQR] and Mann-Whitney U-test.

To gain insight into potential mechanisms underlying the observed contrast-induced effects, we re-examined how the variation of each model parameter influences the area summation curve (Figures 2C–F). Changes in any single parameter cannot reproduce the summation field increase and peak response rate decrease observed when reducing contrast. However, both effects can be simultaneously achieved by changing *pairs* of parameters. One possibility is that stimulus contrast simply regulates both *k*_*D*_ and *k*_*N*_: a gain-specific mechanism. Alternatively, lowering stimulus contrast might be accounted for by a decrease in *k*_*D*_ and an increase in *w*_*D*_: an input-drive–specific mechanism. To distinguish between these and other possibilities, we first compared changes in all parameters estimated for high vs. low contrast during the control condition (Table 3). Lowering contrast caused both gain parameters to decrease (Figures 5E,F) and both size parameters to increase (Figures 5G,H). However, the effect was at least three times larger for the gain parameters than the field diameter parameters: Δ*k*_*D*_ = −73%, Δ*k*_*N*_ = −62%, Δ*w*_*D*_ = 21%, Δ*w*_*N*_ = 9% (Δindicates change from high to low contrast), and the observed effects in gain parameters were more consistent across the population: *k*_*D*_↓ 95% units, *k*_*N*_ ↓ 83% units, *w*_*D*_ ↑ 23% units, *w*_*N*_ ↑ 38% units (arrow indicates direction of parameter change followed by percentage of units). The most likely interpretation is that stimulus contrast primarily engages a gain-specific mechanism of area summation, whereby both the excitatory drive and normalization pool signals scale in proportion to stimulus contrast.

**Figure 7:**
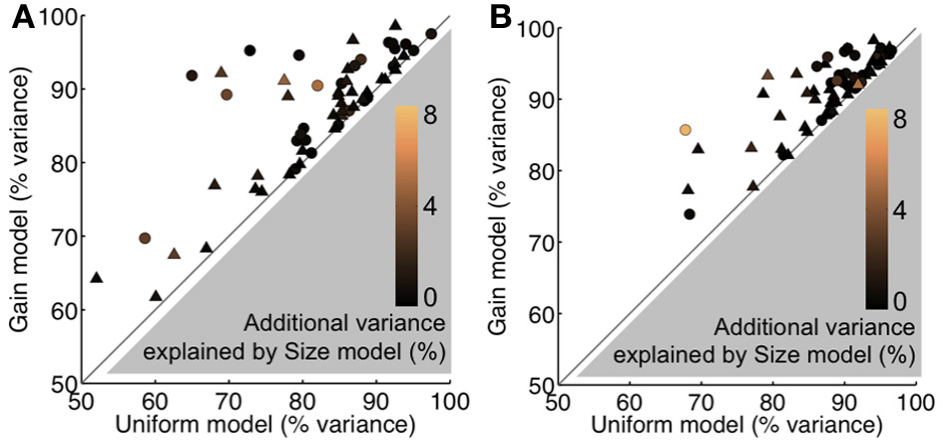
**Constrained model comparisons. (A)** Variance explained per unit during the Control condition, plotted for the *gain* model vs. *uniform* model. Color scale indicates additional variance explained by the *size* model relative to *gain* model. Gray region cannot be occupied because the *gain* model has one additional parameter than the *uniform* model. Similarly, the color scale is non-negative because the *size* model has one additional parameter than the *gain* model. **(B)** Same format as **(A)** for the Feedback inactivation condition. The *gain* model considerably improves the goodness-of-fit, while only slight improvements are observed with the *size* model. For all panels, triangles and circles indicate single- and multi-units, respectively.

To further evaluate the possibility of a contrast-related gain mechanism, we followed the approach of Cavanaugh et al. (2002) by comparing three fixed-parameter versions of the area summation model for each unit. The *uniform* model varies *k*_*D*_ only; the *gain* model varies {*k*_*D*_, *k*_*N*_}, and the *size* model varies {*k*_*D*_, *k*_*N*_, *w*_*D*_}. Each version of the model is rendered more complex than the previous one by allowing an additional parameter to vary across contrast conditions. Note that the sequence of adding parameters here follows the magnitude of the effects described in the previous paragraph when examining changes in individual parameters (Δ*k*_*D*_, Δ*k*_*N*_, Δ*w*_*D*_, Δ*w*_*N*_). Therefore, our approach progressively makes the models more complex by allowing the next best-available parameter to vary across contrast. Model comparisons were again judged in terms of goodness-of-fit, but this time, in addition to percentage of variance explained, we used the χ^2^_N_ statistic in order to account for the differing degrees of freedom across models (see Materials and Methods). In agreement with Cavanaugh et al. (2002), we found that the *gain* model was most efficient in explaining the control data in terms of variance explained (Figure 7A) and χ^2^_N_, the goodness-of-fit per *df* (*uniform* χ^2^_N_ = 5.60; *gain* χ^2^_N_ = 4.78; *size* χ^2^_N_ = 5.13; see Materials and Methods). Therefore, during control conditions, the effects of contrast on area summation were best explained by changes in the gains of the underlying excitatory drive and normalization components, with no need to invoke any changes in their sizes. During feedback inactivation, we again found that the *gain* model was the best at explaining the effect on contrast (*uniform* χ^2^_N_ = 4.65; *gain* χ^2^_N_ = 3.79; *size* χ^2^_N_ = 3.95; Figure 7B). Indeed, fixing *w*_*D*_ and *w*_*N*_ reduced the variance explained by only a few percent, and their feedback-induced parameter shifts were qualitatively similar (Table 4). These results show that the effect of stimulus contrast on area summation in V1, as well as the underlying gain-related mechanism, remains largely intact in the absence of feedback from V2 and V3.

**Table 4:** **Comparison of area summation model and gain model**.

	**Area summation model**		**Gain model**	
**Free across contrast:**	***k_D_, k_N_, w_D_, w_N_***		***k_D_, k_N_***	
**Control condition**	**High contrast**	**Low contrast**	**High contrast**	**Low contrast**
Peak size (°)	0.46 ± 0.20	0.71 ± 0.65	0.47 ± 0.19	0.61 ± 0.26
Peak rate (s^−1^)	54 ± 32	32 ± 22	54 ± 32	31 ± 21
Asymptote size (°)	4.4 ± 1.5	4.6 ± 1.6	4.3 ± 1.5	4.2 ± 1.6
Asymptote rate (s^−1^)	12 ± 15	9.6 ± 12	12 ± 16	11 ± 13
SSI (a.u.)	0.79 ± 0.17	0.71 ± 0.26	0.78 ± 0.19	0.67 ± 0.27
*k*_*D*_ (a.u.)	1730 [3110]	532 [613]	1720 [3220]	600 [636]
*k*_*N*_ (a.u.)	12.9 [13.8]	5.02 [5.89]	13.6 [16.5]	4.59 [5.27]
*w*_*D*_ (°)	0.32 ± 0.25	0.39 ± 0.29	0.34 ± 0.17 (fixed)	
*w*_*N*_ (°)	1.96 ± 1.29	2.14 ± 1.31	1.79 ± 1.19 (fixed)	
Normalized χ^2^	5.68		4.78	
Variance explained (%)	88%		87%	
**Feedback inactive**	**High contrast**	**Low contrast**	**High contrast**	**Low contrast**
Peak size (°)	0.51 ± 0.22	0.72 ± 0.38	0.52 ± 0.22	0.65 ± 0.32
Peak rate (s^−1^)	59 ± 35	37 ± 27	59 ± 35	36 ± 27
Asymptote size (°)	4.1 ± 1.6	4.7 ± 1.5	4.1 ± 1.5	4.1 ± 1.6
Asymptote rate (s^−1^)	19 ± 20	12 ± 14	19 ± 20	14 ± 15
SSI (a.u.)	0.71 ± 0.22	0.68 ± 0.25	0.71 ± 0.22	0.61 ± 0.27
*k*_*D*_ (a.u.)	1770 [2440]	450 [817]	1830 [2290]	509 [857]
*k*_*N*_ (a.u.)	7.99 [14.5]	3.79 [5.82]	10.2 [14.9]	3.80 [4.98]
*w*_*D*_ (°)	0.31 ± 0.18	0.40 ± 0.20	0.36 ± 0.22 (fixed)	
*w*_*N*_ (°)	1.72 ± 1.22	2.11 ± 1.33	1.36 ± 1.07 (fixed)	
Normalized χ^2^	4.32		3.79	
Variance explained (%)	92%		90%	

Mean ± SD used everywhere except for k_D_ and k_N_ reported as median [IQR]. Some results from Table 1 are repeated here for convenience.

### Center contrast sensitivity unaltered during feedback inactivation

**Figure 8:**
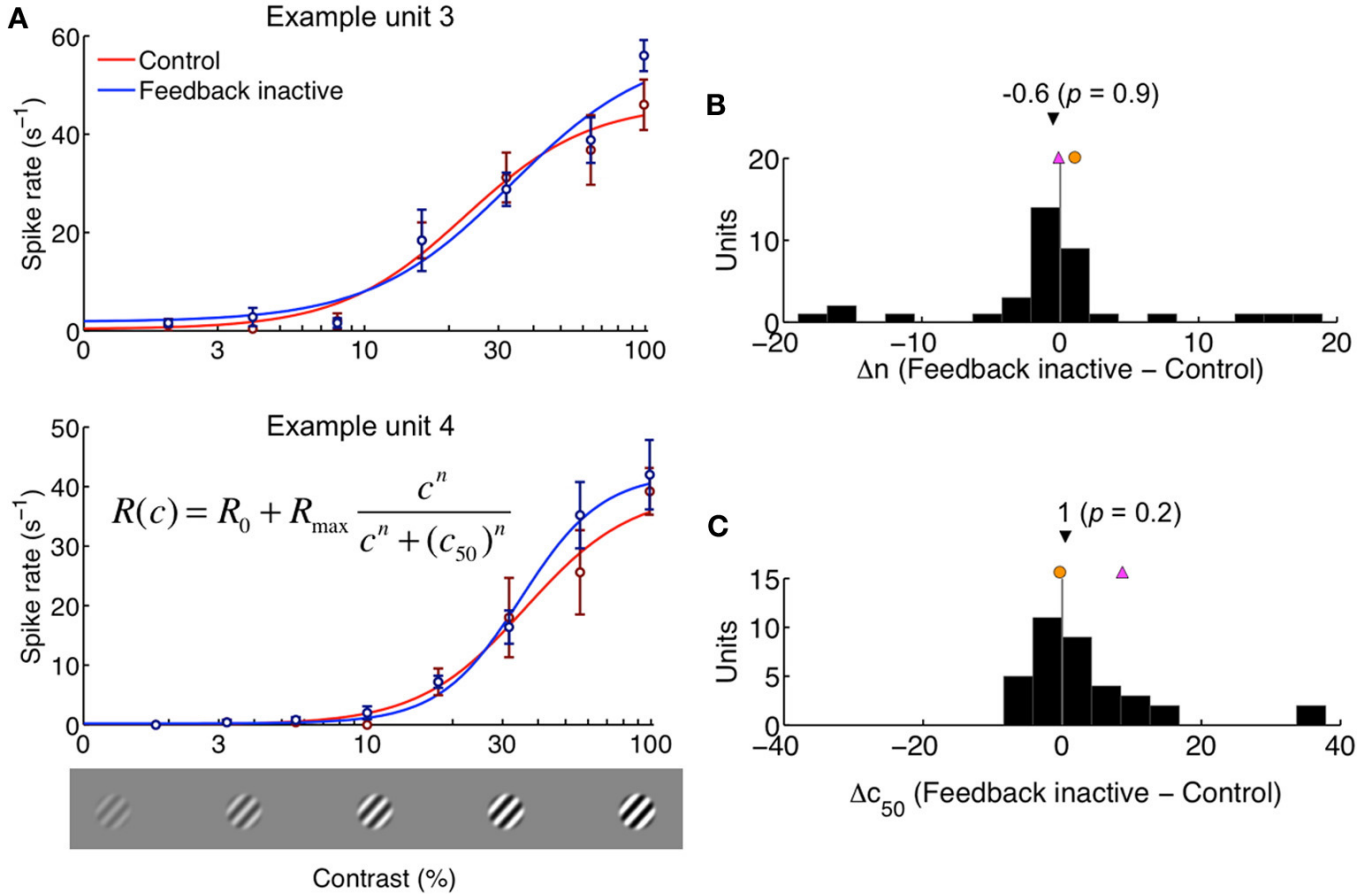
**Contrast sensitivity is unaltered by feedback inactivation. (A)** Contrast response functions plotted for two example units. The grating was restricted to the center of the receptive field. Data are shown as mean ± s.e.m., and curves were obtained using the Naka-Rushton model (inset equation) by fitting the parameters *n* and *c*_50_. The spike rates for spontaneous activity (R_0_) and maximum response (R_max_) were measured. **(B)** Difference in parameter *n* between Feedback inactive and Control conditions. **(C)** Difference in parameter *c*_50_ between Feedback inactive and Control conditions. Black triangle indicates population median (with two-tailed Mann-Whitney *U*-test *p*-value). Magenta and orange symbols indicate shifts for example units shown in **(A)**. Feedback-inactivation did not lead to a systematic effect in contrast response function parameters.

The above results show that feedback inactivation had little or no effect on the gains of the underlying excitatory drive and normalization pool components. If this is indeed the case, we would not expect to observe population-wide changes in contrast sensitivity for small stimuli restricted to the receptive field center. We measured the contrast response function for 36 units from the same two monkeys (24 single units, 12 multi-units). The experimental protocol was identical to that used for area summation measurements, except that gratings were fixed in size, appeared entirely within the experimentally-derived high contrast summation field, and eight contrast values were pseudo-randomly presented. All 36 units were sensitive to contrast (Two-Way ANOVA, main effect contrast, each *p* < 0.05), and the relationship between contrast and neural responses was a roughly sigmoidal, monotonic-increasing function (Figure 8A). Although 17 of 36 units demonstrated some sensitivity to feedback, the effects were weak and variable and thus did not result in significant effects at the population level (Two-Way ANOVA, main effect of feedback and contrast × feedback inactivation, both *p* > 0.05).

We fit each unit's contrast sensitivity curve with the Naka-Rushton hyperbolic ratio function (see Materials and Methods), which explained on average over 90% of the variance across the population. We found no trends for systematic differences across feedback conditions for either the exponent parameter *n* or the *c*_50_ parameter (Mann-Whitney *U*-test, both *p* > 0.2; Figures 8B,C). In addition, we did not detect a change in contrast response saturation across the population (*R*_max_: 52 ± 35 s^−1^ vs. 52 ± 32 s^−1^, feedback on vs. off respectively, *p* = 0.86). Despite the lack of evidence for a contrast gain mechanism contributing to the effects of feedback inactivation on area summation, we tested for a correlation between changes in either *n* or *c*_50_ and the corresponding feedback-related changes in surround suppression (ΔSSI from the area summation data) and found no correlation in either case: *r*(ΔSSI, Δ*n*) = 0.03 and *r*(ΔSSI, Δ*c*_50_) = −0.22 (both *p* > 0.1). The absence of a systematic influence of feedback inactivation on contrast sensitivity within the high contrast summation field provides a second line of evidence—consistent with the area summation analysis—that feedback does not influence area summation properties in V1 *via* a contrast-gain mechanism.

## Discussion

Our results suggest that feedback from V2 and V3 contributes to area summation properties in V1 through normalization. During feedback inactivation, we observed increased responses in V1 to large stimuli, with little or no effect on responses to small stimuli, leading to a reduction in the strength of surround suppression (Figure 3). The effects of V2/V3 cooling on the area summation curves were well explained by a normalization model in which feedback inactivation reduced the spatial extent of a divisive normalization pool (Figures 2, 3). Contrast-dependent effects on area summation were unaltered during feedback inactivation (Figure 6), as was contrast sensitivity within the receptive field center (Figure 8), providing evidence that feedback acts independently of previously described contrast-gain mechanisms known to exist in V1 (Sclar et al., 1990; Levitt and Lund, 1997; Cavanaugh et al., 2002).

The original normalization model (Heeger, 1992) was put forward to account for two shortcomings of the Hubel-Wiesel linear model of simple cells (Hubel and Wiesel, 1962): (1) response saturation at high contrast and (2) non-specific suppression of responses due to additional stimuli in or near the classical receptive field. Although the normalization model accounts for both processes with the same equation, recent evidence indicates that the two may in fact be mechanistically distinct (Olsen et al., 2010): Working in the olfactory system of Drosophila, these investigators demonstrated that response saturation occurred within each glomerular channel, while normalization was due to lateral inhibitory interactions between glomeruli. Our results show a similar dissociation. We found that feedback inactivation reduced surround suppression solely by shrinking the spatial extent of the normalization pool and, remarkably, that this effect was independent of the gain modulations that accounted for effects of stimulus contrast on area summation. We further confirmed that contrast sensitivity within the classical receptive field is unaltered when inactivating feedback. Insofar as divisive normalization underlies area summation, our results provide direct evidence that corticocortical feedback participates in normalization independent of the circuit mechanisms involved in contrast-gain control. It is nevertheless important to note that we applied normalization as a premise for interpreting these data. Therefore, our results do not rule out alternative models that account for summation properties (e.g., Sceniak et al., 1999).

Based on our data, feedback from V2 and V3 interacts preferentially with the pool of neurons that produces surround suppression in V1 rather than the pool that provides the excitatory drive. This is consistent with earlier propositions that feedback acts as a modulator of neural activity as opposed to a driver (Shao and Burkhalter, 1996; Sherman and Guillery, 1998; Angelucci and Bressloff, 2006). Such a characterization is based largely on anatomical work which has shown that feedback axons tend to target the distal apical dendrites of pyramidal neurons (Rockland and Virga, 1989; Anderson and Martin, 2009) and are therefore capable of having an impact on the gain of neuronal outputs (Larkum et al., 2004). However, here we have provided evidence contrary to the idea that feedback modulates gain, but rather that it sets the size of the suppressive field. The idea that feedback's role as a modulator can be *suppressive* has not been fully appreciated until recently (Nassi et al., 2013). Previously, one of the only clues regarding feedback's involvement in area summation was the relatively fast response *facilitation* from far regions of the receptive field surround (Ichida et al., 2007). Surround suppression could be mediated by feedback connections directly onto local inhibitory neurons (Gonchar and Burkhalter, 2003; Anderson and Martin, 2009). This would be consistent with recent work in the rodent, showing that somatostatin-positive inhibitory neurons play an important role in surround suppression (Adesnik et al., 2012) and that long range excitatory projections can have a net suppressive effect through the recruitment of local inhibitory neurons (Iurilli et al., 2012; Palmer et al., 2012). Suppression through feedback could also be mediated indirectly via horizontally projecting excitatory neurons in superficial layers which are known to target inhibitory neurons (McGuire et al., 1991) and have been implicated in several forms of “top-down” modulatory influences (Ito and Gilbert, 1999; Li et al., 2004; Ramalingam et al., 2013). Feedback need not rely solely on inhibitory neurons in order to produce suppression, as increases in conductance as well as synaptic depression can lead to reduced neuronal output without a net increase in inhibition. In fact, recent evidence suggests that surround suppression relies on the dynamic interplay between highly interconnected excitatory and inhibitory networks, such as an inhibition-stabilized network, and there are likely to be many ways by which feedback could modulate such a balanced network regime (Ozeki et al., 2009; Haider et al., 2010).

In distinction to inputs from the normalization pool, driving inputs received by V1 neurons are thought to establish basic selectivity for such properties as spatial position and orientation, and are likely mediated primarily by feed-forward connections (Priebe and Ferster, 2012). In support of this idea is our previously reported observation that orientation preference within the receptive field center is unaffected during feedback inactivation (Nassi et al., 2013). However, we did find that selectivity for orientation was often reduced due to slightly increased responses to orthogonal orientations when feedback was inactivated. This is consistent with models of orientation tuning that depend on local cortical circuits to sharpen orientation preference (Ben-Yishai et al., 1995; Somers et al., 1995; Troyer et al., 1998); an operation that may also rely on normalization (Heeger, 1992).

According to our modeling results, feedback from V2 and V3 contributes to the overall size but not the gain of normalization in V1. Specifically, feedback increases the spatial extent over which normalization operates. An increase in the size of the normalization pool leads to increased suppression in the surround while leaving responses within the receptive field center unchanged (Figure 2F). This is consistent with both the area summation data and center contrast response function data presented here (Figures 1, 8), as well as the fact that receptive fields increase in size along the visual cortical hierarchy. Feedback from V2 and V3 can cover aggregate visual fields five–ten times the size of the receptive field center in V1 (Angelucci and Bressloff, 2006). This may explain why observed effects of feedback inactivation were strongest for stimulus diameters approximately two–eight times the size of the receptive field center and relatively weaker for the largest stimulus diameters tested (Nassi et al., 2013), which likely evoked stronger responses from areas such as MT that remained active during cooling of V2/V3 (Ponce et al., 2008). Indeed, substantial suppression remained during feedback inactivation, not only for the largest stimulus diameters tested, but also for stimulus diameters that extended just beyond the center of the receptive field. These results suggest that, in addition to feedback from V2/V3, other cortical sources of feedback, as well as horizontal connections intrinsic to V1, all combine to produce the level of surround suppression observed under normal conditions. Each of these distinct sources of suppression likely operates at different spatio-temporal scales (Bair et al., 2003; Angelucci and Bressloff, 2006; Reynaud et al., 2012) and their interaction with one another within the context of normalization will be an important question to be addressed in the course of future research.

Area summation properties are not only dependent on top-down feedback signals but also on bottom-up stimulus properties such as luminance contrast (Levitt and Lund, 1997). As stimulus contrast is reduced, the size of the summation field increases. This was first accounted for with a difference of Gaussians model in which a reduction in contrast increases the spatial extent of excitatory drive (Sceniak et al., 1999). In subsequent work, it was shown that the increase in summation field size at low contrast could be more parsimoniously explained by a divisive normalization model in which input drive and the normalization pool are stable in spatial extent and only their *relative gains* depend on contrast (Cavanaugh et al., 2002). Our analysis reproduced the contrast gain results of the Cavanaugh model (Figure 7). The current results further showed that inactivation of feedback leaves contrast-gain effects on area summation unaltered (Figure 6). This suggests that the effects of contrast on area summation are mediated by circuit mechanisms independent of feedback. One likely candidate would be horizontal connections intrinsic to V1, which have been shown to cover the necessary visuotopic extent (Angelucci and Bressloff, 2006). Indeed, recent evidence from voltage-sensitive dye imaging in alert macaques suggests that horizontal connections may act to dynamically clamp local contrast gain mechanisms (Reynaud et al., 2012). Interestingly, these investigators were able to account for their observed results with two distinct normalization processes, proposing that recurrent polysynaptic intracortical loops mediate contrast gain and long-range monosynaptic horizontal spread mediates surround suppression. Taken together, our results suggest that horizontal connections in V1 may set the gain of normalization in V1, whereas feedback sets the extent over which horizontal connections are effective—essentially gating horizontal signals depending on stimulus conditions and behavioral state (Gilbert and Sigman, 2007). This view is consistent with the idea that feedback can modify the “association field” that is likely comprised of local horizontal inputs (Field et al., 1993; Ramalingam et al., 2013).

Recent studies on attention have provided increasing support for the idea that feedback modulates properties of spatial integration (Roberts et al., 2007; Anton-Erxleben et al., 2009; Sundberg et al., 2009). For example, in V4 attention directed to the receptive field center weakened suppression from the surround, while attention to the surround enhanced suppression (Sundberg et al., 2009). These effects were accounted for by a normalization model similar to the one considered here (Lee and Maunsell, 2009; Reynolds and Heeger, 2009). In V1, effects of attention have been found to be more complicated: some studies have found that attention reduces the impact of the surround, whereas others have found that the impact of the surround is enhanced (Ito and Gilbert, 1999; Roberts et al., 2007; Chen et al., 2008; Poort et al., 2012). These effects appear to depend on several factors including stimulus eccentricity, whether surround stimuli are facilitatory or suppressive and the nature of the perceptual task. In the current study, the behavioral task was simply to fixate a central cross, leaving the attentional focus of the animals uncontrolled and thus indeterminate. It is probable, however, that the receptive fields we studied fell outside of the locus of attention which was most likely directed toward the fixation point—a configuration for which suppressive effects are expected. A prediction for future studies is that training the animal to direct attention toward the receptive fields under study might uncover excitatory influences of feedback.

### Conflict of interest statement

The authors declare that the research was conducted in the absence of any commercial or financial relationships that could be construed as a potential conflict of interest.
