# Supplementary figures and images for: Corticocortical feedback increases the spatial extent of normalization
Source: Front Syst Neurosci. 2014 May 30;8:105. doi: 10.3389/fnsys.2014.00105 (PMC4039070; doi:10.3389/fnsys.2014.00105)

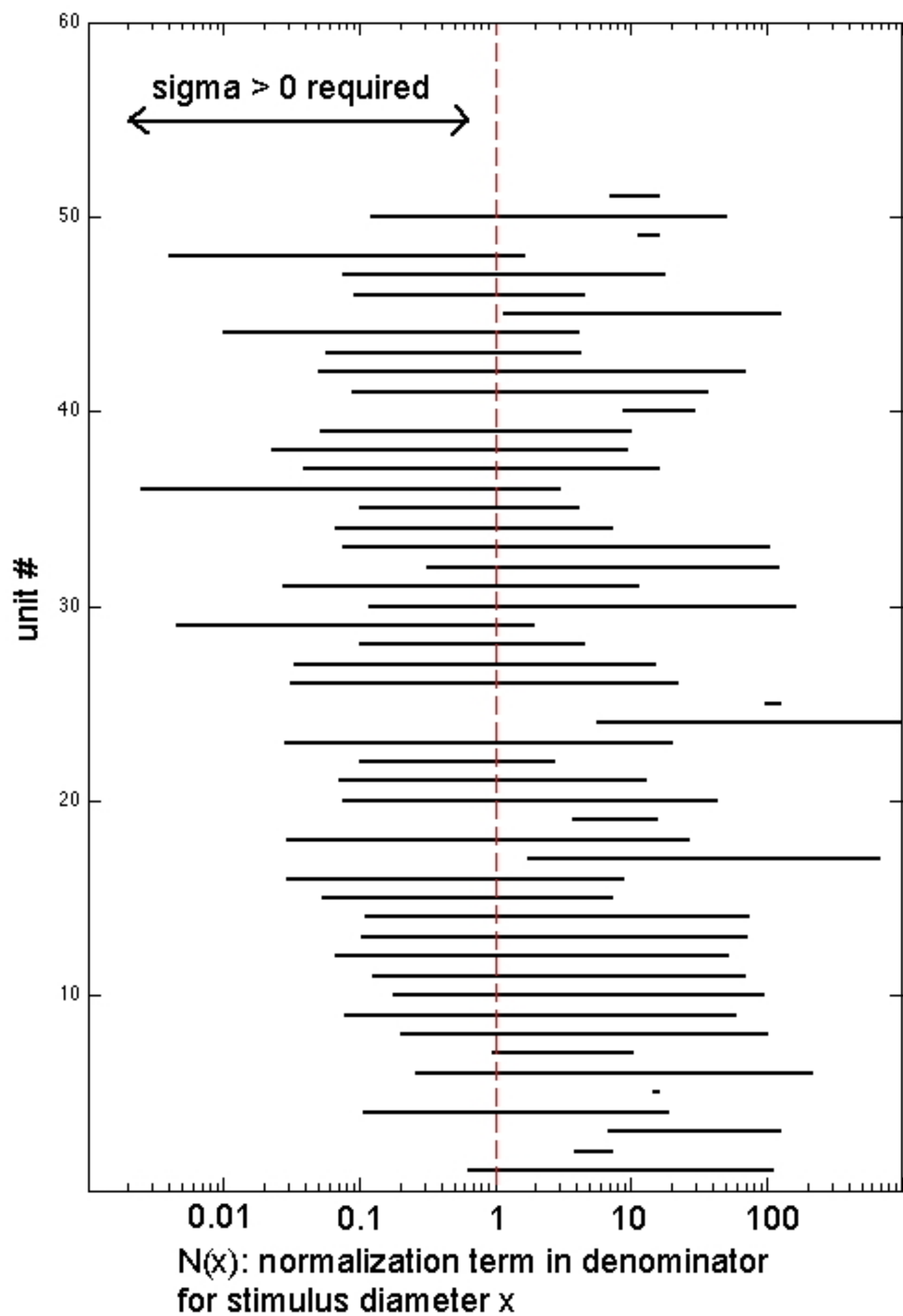

Supplement: Supplementary file 1 [file Presentation1.PDF]
